# Use of Microbial Fuel Cells for the Treatment of Residue Effluents Discharged from an Anaerobic Digester Treating Food Wastes

**DOI:** 10.3390/microorganisms11030598

**Published:** 2023-02-27

**Authors:** Daichi Yoshizu, Atsushi Kouzuma, Kazuya Watanabe

**Affiliations:** Laboratory of Bioenergy Science and Technology, School of Life Sciences, Tokyo University of Pharmacy and Life Sciences, Hachioji, Tokyo 192-0392, Japan

**Keywords:** anaerobic digester, microbial fuel cell, total nitrogen, total phosphorus, hydraulic residence time

## Abstract

One of practical challenges in anaerobic-digestion (AD) technology is the cost-effective treatment of residue effluents containing high concentrations of organics, nitrogen and phosphorus (CNP). In order to evaluate the utility of microbial fuel cells (MFCs) for treating anaerobic-digester effluents (ADEs) and generating power from them, laboratory-scale single-chamber MFCs were filled with ADE obtained from a commercial AD plant treating food wastes and thereafter operated by routinely supplying ADE at different hydraulic residence times (HRTs, 5 to 20 days). It is shown that MFCs were able to reduce not only organics in ADE but also nitrogen and phosphorus. For instance, data demonstrated that over 50% of CNP was removed in MFCs operated at an HRT of 10 days, at which the maximum power density reached over 200 mW m^−2^ (based on the projected area of anode). Metabarcoding of 16S rRNA genes showed that some bacteria were specifically enriched in anode biofilms, suggesting their involvement in power generation. Our study suggests that MFCs are applicable to reducing CNP in ADEs at reasonable rates, and provides subsequent work with fundamental data useful for setting targets for further developments.

## 1. Introduction

Anaerobic digesters (ADs) have widely been used for the treatment of and energy recovery from biomass wastes, such as food wastes [1], livestock manures [2] and sewage sludges [3]. In ADs, microbes degrade biomass wastes and produce methane that can be used as energy for heating and/or electricity generation [4]. Although ADs are able to remove large fractions of organic matter from biomass-waste slurries, residue effluents discharged from ADs (AD effluents, ADEs) still contain substantial amounts of organics, nitrogen and phosphorus. For instance, concentrations of organics in ADEs typically exceed 10,000 mg L^−1^, as measured by biological oxygen demands (BODs) and/or chemical oxygen demands (CODs) [5]. ADEs, in particular those rich in nitrogen, can be used as fertilizers in agricultural areas [6], while in urban areas ADEs have to be further treated to decrease BODs/CODs, total nitrogen (TN) and total phosphorus (TP) down to levels at which these are permitted to be discharged into sewers [7]. For treating ADEs, aerobic processes such as activated-sludge tanks and/or aerobic membrane bioreactors are used [7], and substantial amounts of cost and energy are needed for the treatment.

Microbial fuel cells (MFCs) are devices that exploit microbes for the conversion of organic matter into electric energy [8]. MFCs are primarily expected to be applied to energy recovery from biomass waste and wastewater [9], while researchers have suggested additional merits of MFCs over aerobic processes, such as no need of aeration and low sludge production [10]. MFC reactors suited to the treatment of biomass wastes have been designed, and their utilities have been demonstrated in the laboratory [11]. Several studies have also examined MFCs for the treatment of and energy recovery from ADEs [12,13,14,15]. To cite an instance, Inglesby and Fisher [12] have attempted to apply MFC to the polishing of ADE from an anaerobic digester treating *Arthrospira maxima* biomass. In addition, Cerrillo et al. [14] have investigated a four-module tubular MFC for recovering energy from filtered effluents from a two-stage biohydrogen and biomethane system, and reported a volumetric power density of 3.1 W m^−3^. Although these studies have reported electricity generation and organics removal in MFCs treating ADEs, information regarding the removal of TN and TP from ADEs has not been reported.

The present study was conducted to evaluate the MFC technology for its application to the treatment of ADE discharged from a commercial plant treating food wastes. A particular focus was placed on how the hydraulic residence time (HRT) affects MFC performances, and this study examined TN and TP removal in addition to organics removal and electricity generation. Furthermore, given that soluble fractions of ADE are treated in downstream wastewater-treatment facilities, COD, TN and TP in soluble fraction of MFC effluents (MFCE) were also measured.

## 2. Materials and Methods

### 2.1. ADE

The ADE used in this work was obtained from a commercial plant treating food wastes. A typical total COD (tCOD) was approximately 40,000 mg L^−1^, while soluble COD (sCOD, COD for supernatant after centrifugation of ADE at 15,000 × *g* for 5 min) was approximately 5000 mg L^−1^.

### 2.2. MFC Setups

The MFCs used in this work were single-chamber reactors. The volume of the chamber was 85 mL. A reactor was equipped with a single pair of anode and cathode, and an anode was formed of a graphite felt (20 cm^2^ in area, 5 mm in thickness, Kureca felt, Kureha corporation, Tokyo). For cathodes, this study used gas diffusion-type air cathodes (25 cm^2^) that were formed as described elsewhere [16] and had four polytetrafluoroethylene layers and 0.64 mg cm^−2^ of platinum catalyst (TEC10V20E, Tanaka Kikinzoku Kogyo, Tokyo, Japan). The anode and cathode were connected to an external resister (*R*_ext_, Ω) using titanium and copper wires, and a voltage across *R*_ext_ (*E*, V) was measured using a data logger (GL820, Graphtec, Yokohama, Japan). The ADE in the MFC was gently agitated using a magnetic stirrer at approximately 100 rpm.

### 2.3. Operation of MFCs

Operation of the MFC was initiated by filling it with ADE, and an initial *R*_ext_ was set at 2000 Ω. No other source of microbes was added. During the operation, a part of the liquid in MFC (MFCE) was removed every day, and an equal amount of ADE was added. The amount of MFCE removed from MFC was determined based on HRT, and it was set at 5, 10 or 20 days. Triplicate MFCs were operated under the same HRT condition. After performances of MFCs became stable at 2000 Ω, *R*_ext_ was changed to 500 Ω. Pure water was occasionally added to compensate for water loss from the air cathodes.

### 2.4. Analyses of Power Outputs from MFCs

Current *(I*, mA) was calculated using *E* and *R*_ext_ according to the Ohm’s law (*E* = *I* × *R*_ext_), while power was calculated using *E* and *I* (*E* × *I*) [9]. Current and power densities (*J*, mA m^−2^ and *P*, mW m^−2^, respectively) were calculated based on a projected area of anode (20 cm^2^). Polarization analysis of MFC was conducted using a potentiostat (HSV-110, Hokuto Denko, Tokyo, Japan) at a scan rate of 1 mV s^−1^, and power curves (*J* vs. *P*) were generated based on polarization curves (*J* vs. *E*) [8]. The maximum value of *P* in a power curve was termed *P*_max_ (mW m^−2^), representing the potential of MFC. Coulombic efficiency (*C*_E_, %) was calculated based on tCOD removal (tCOD in MFCE was subtracted from tCOD in ADE) and the measured current using 1 g of COD = 0.125 mol of electron; 1 A = 5.39 × 10^23^ electrons per day [8].

### 2.5. Measurement of COD, TN and TP

ADE and MFCE were used for the measurements of tCOD, total TN (tTN) and total TP (tTP). They were centrifuged at 15,000 × *g* for 5 min, and supernatant fractions were used for measurements of sCOD, soluble TN (sTN), and soluble TP (sTP). COD was measured using TNT823 ultra-high-range (250–15,000 mg L^−1^) system (Hach Co., Loveland, CO, USA), after a sample was appropriately diluted. TN and TP were measured using TNT828 ultra-high-range (Hach Co.) and TNT843 low-range (Hach Co.), respectively, after a sample was appropriately diluted. Organics-loading rates (OLRs, mg L^−1^ day^−1^) were calculated from tCOD in ADE and HRT [9].

### 2.6. Metabarcoding of Anode Biofilms and Planktonic Microbiomes

DNA was extracted from anode biofilms (ABs) and planktonic microbiomes (PMs) in MFCs using FastDNA SPIN kit for soil (MP Biomedicals, Irvine, CA, USA). A small piece of anode (approximately 1.0 cm^2^) was cut and put in a tube of the kit for DNA extraction from AB. Approximately 800 µL of MFCE was added to a tube of the kit for DNA extraction from PM. The V3/V4 region in 16S rRNA genes was amplified from extracted DNA using primers described elsewhere [17]. Amplification of DNA fragments was confirmed by electrophoresis. PCR products were purified using a QIAquick PCR purification kit (Qiagen, Venlo, The Netherlands), and, after DNA concentrations were determined by measuring absorption spectra using a spectrophotometer (Nano-drop, Thermo Scientific, Waltham, MA, USA), amplicon samples were mixed at a same concentration and subjected to pair-end sequencing using a MiSeq sequencer (Illumina, San Diego, CA, USA) according to a protocol recommended by the manufacturer. Sequence reads greater than 230 bp were collected, and chimeric sequences were detected and removed using USEARCH via the uchime command [18]. Sequences were clustered into operational taxonomic units with 97% similarity using QIIME 2 [19] and taxonomically classified by aligning these values with sequences in the Greengenes database [20]. The sequences generated in the metabarcoding analysis were deposited in the DDBJ Sequence Read Archive database under accession number DRA015529.

## 3. Results

### 3.1. Power Generation from ADE

The MFCs were operated by replacing parts of MFCE with ADE every day, and HRTs were set at 5, 10, or 20 days by changing volumes of MFCE replaced with ADE at one time. We employed these HRT values, since the commercial AD plant from which we obtained ADE was operated at a HRT of approximately 20 days, and it would be desirable that the MFC used to treat the ADE was smaller than the AD. As the liquid in the MFC was agitated not to precipitate sludge and solids, sludge residence time was the same as HRT. OLRs for MFCs at HRTs of 5, 10 and 20 days were approximately 8000, 4000 and 2000 mg L^−1^ day^−1^, respectively. We initiated the operation of MFCs using microbes in ADE without inoculating with other microbial sources, since previous studies have successfully used AD sludge as inocula for MFCs [21,22].

**Figure 1:**
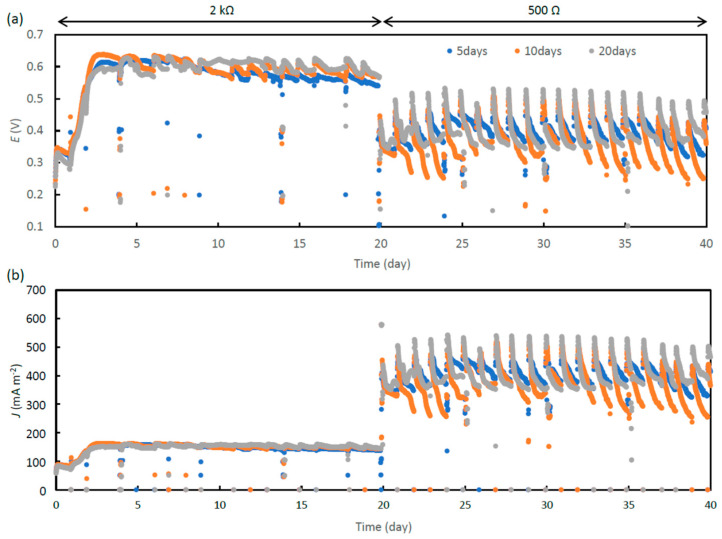
Representative curves showing changes in *E* (**a**), and *J* (**b**), for ADE-treating MFCs operated at HRTs of 5, 10 and 20 days. *R*_ext_ was changed from 2000 Ω to 500 Ω on day 20.

The MFCs were initially operated at *R*_ext_ of 2000 Ω, and changed to 500 Ω on day 20. Figure 1a,b shows the changes in *E* and *J* during the operation. As shown in this figure, *E* was stably high at around 0.6 V, when *R*_ext_ was 2000 Ω. However, it fluctuated between 0.3 V and 0.5 V, when *R*_ext_ was 500 Ω. At 500 Ω, the addition of ADE to MFC resulted in the immediate increase in *E*, which then gradually decreased. This trend was considered to be attributable to the fact that larger currents were generated in MFCs at 500 Ω than those at 2000 Ω (Figure 1b), suggesting that larger amounts of organics, particularly those directly used for current generation, were removed at 500 Ω (refer to the subsequent sections for organics removal at 500 Ω).

**Figure 2:**
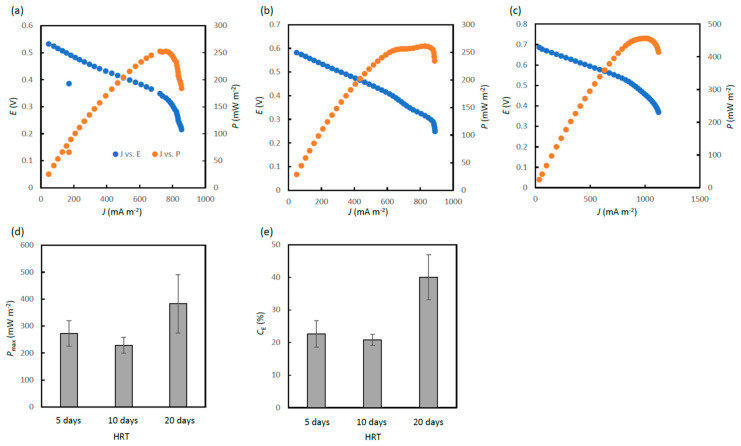
Power generation by ADE-treating MFCs operated at HRTs of 5, 10 or 20 days. Representative polarization and power curves for MFCs operated at HRTs of 5, 10 and 20 days are shown in (**a**), (**b**), and (**c**), respectively. (**d**) Comparison of *P*_max_ values for MFCs operated at the different HRTs (*R*_ext_ was 500 Ω). (**e**) Comparison of *C*_E_ values for MFCs operated at the different HRTs (*R*_ext_ was 500 Ω). In (**d**) and (**e**), mean values and standard errors are shown (n = 9).

Polarization analyses were conducted to determine *P*_max_, the potential of power generation, for MFCs operated at the different HRTs, and representative polarization and power curves are presented in (a), (b) and (c) in Figure 2. From *J* and *E* values at which *P*_max_ values were given in power curves, the optimum *R*_ext_ (*R*_ext_ that generates the highest power) could be estimated, and this was 313, 375 and 350 Ω for MFCs operated at HRTs of 5, 10 and 20 days, respectively. From these estimates, it was considered that *R*_ext_ of 500 Ω was close to the optimum for MFCs used in this study for the treatment of ADE. We, therefore, decided not to further decrease *R*_ext_ in our experiments.

During the operation of MFCs at *R*_ext_ of 500 Ω, polarization analyses were conducted three times (days 30, 35 and 40), and *P*_max_ values were determined. Mean values of *P*_max_ for MFCs at the different HRTs are compared in Figure 2d. As indicated in this figure, the *P*_max_ values for MFCs operated at HRTs of 5 and 10 days were not significantly different, while the *P*_max_ for MFCs at an HRT of 20 days was substantially higher than the others. This result was unexpected, and we discuss this result in the discussion section.

In the present work, *C*_E_, an index that shows the efficiency of electron recovery as current in MFC, was also estimated on days 30, 35 and 40. *C*_E_ values for MFCs operated at HRTs of 5, 10 and 20 days are compared in Figure 2e. It was shown that *C*_E_ values for MFCs operated at HRTs of 5 and 10 days were not significantly different, while the *C*_E_ values for MFCs at an HRT of 20 days was substantially higher than the others. The high *C*_E_ value for MFCs at an HRT of 20 days was considered reasonable, since this value was calculated from *I* and tCOD removal, and values for the tCOD removal were low at low OLRs (e.g., those at an HRT of 20 days).

### 3.2. Removal of COD, TN and TP from ADE

In order to evaluate if MFC is able to reduce COD, TN and TP in ADE, those in MFCE were measured and compared with those in ADE obtained from the AD plant. In the AD plant from which ADE was obtained, ADE is dehydrated by centrifugation, and liquid fraction is treated in a wastewater-treatment facility before discharge into the sewer. On the other hand, solid fraction is partly used as fertilizer, and the rest is incinerated. It is, therefore, important to reduce TN and TP, in addition to COD, in the soluble fraction of ADE, for the stable treatment of ADE and discharge of soluble fractions into the sewer. In the present work, in addition to tCOD, tTN and tTP, sCOD, sTN and sTP were, therefore, measured.

**Figure 3:**
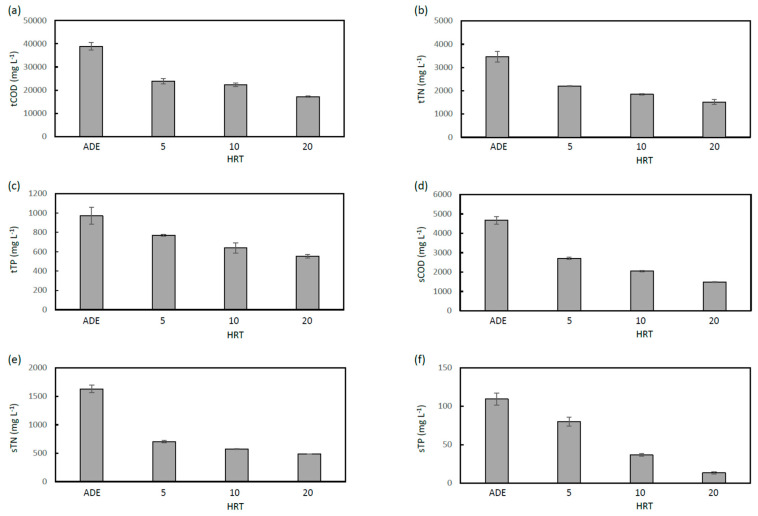
Total and soluble COD, TN and TP in MFCE from MFCs operated at HRTs of 5, 10 and 20 days. Data of tCOD (**a**), tTN (**b**), tTP (**c**), sCOD (**d**), sTN (**e**) and sTP (**f**) for MFCE removed from respective MFCs on day 40 are shown. These values for ADE are also presented in the graphs. Mean values and standard errors are shown (n = 3).

This study measured COD, TN and TP in the total and soluble fractions of MFCE removed from MFCs on day 30, 35 and 40, when *R*_ext_ was 500 Ω, showing that daily variations of these values were not significant (data not shown). Data of tCOD, tTN, tTP, sCOD, sTN and sTP on day 40 for MFCs operated at HRTs of 5, 10 and 20 days are collectively presented in Figure 3. As shown in these graphs, all these values were decreased by the MFC treatment independent of HRT, while the lowest values were observed at HRT of 20 days for all indices. In addition, the data show that larger ratios of decreases in these values were observed for the soluble fractions than those for the total fractions.

The removal of COD was affected by HRT, while its influence on the soluble fraction was generally larger than that on the total fraction (Figure 3a,d). As indicated in Figure 2e, approximately 40% of the tCOD removal was ascribable to the current generation at an HRT of 20 days, while approximately 20% was ascribable to those at HRTs of 5 and 10 days. The rests were considered to be associated with respiration using oxygen that invades through air cathodes and/or methanogenesis [11].

The present study also found that TN and TP in ADE were decreased by the MFC treatment. As shown in Figure 3b,e, tTN decreased gradually depending on HRT, while sTN substantially decreased at an HRT of 5 days, and further decrease was small at the other HRT values. These data suggest that nitrogen compounds (approximately 500 mg L^−1^) would exist that are hardly removed from ADE by the MFC treatment. It was shown that MFC was able to reduce sTN down to approximately one third (from 1500 mg L^−1^ to 500 mg L^−1^) of that in ADE.

Compared with the decreases in tTN, those in tTP were not large despite that the absolute value of tTP in ADE was smaller than that of tTN (Figure 3c). As shown in Figure 3f, however, substantial decreases in sTP were observed, and the decrease was largely dependent on HRT. The substantial decrease in sTP by the MFC treatment was unexpected, while it was likely that sTP was mostly taken up by microbes and became insoluble, which HRT affects. Mechanisms for the decreases in TN and TP are further discussed in the discussion section.

### 3.3. Metabarcoding of Bacteria in the AB and PM Fractions

**Figure 4:**
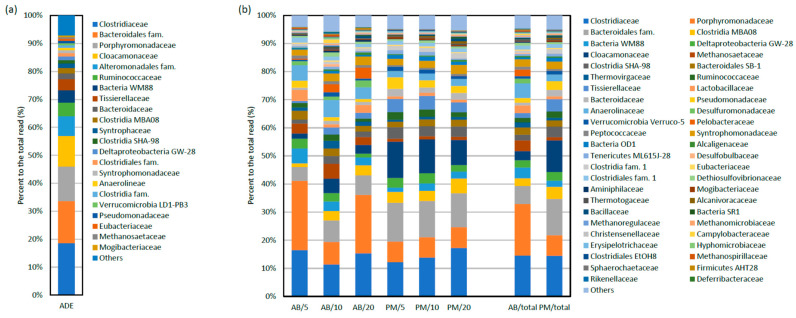
Comparative metabarcoding for bacteria in MFCs operated at different HRTs. (**a**) A metabarcoding profile for ADE obtained from the AD plant. (**b**) Metabarcoding profiles for bacteria in MFCs. Sample names indicate anode biofilms (AB), planktonic microbes (PM) and HRT of MFCs (5, 10 and 20 days). Sums of metabarcoding data for the AB fractions in MFCs at HRTs of 5, 10 and 20 days and those for the PM fractions are presented as AB/total and PM/total, respectively.

In order to gain insights into bacteria that are involved in the generation of power and removal of COD, TN and TP in MFCs treating ADE, metabarcoding of 16S rRNA gene fragments amplified by PCR from ADE obtained from the AD plant and those from the AB and PM fractions in MFCs was conducted, and the results are compared at the family level in Figure 4. Figure 4a shows a result for ADE obtained from the AD plant. In Figure 4b, in addition to metabarcoding results for the AB and PM fractions in MFCs operated at HRTs of 5, 10 and 20 days, those for the total AB and PM fractions (a sum of data for each bacterial family in MFCs operated at the different HRTs) are also shown. In these analyses, although we used bacteria-specific primers for the amplification of 16S rRNA gene fragments, some archaeal families were also detected. Given the importance of methanogenic archaea in AD, it is likely that actual archaeal populations in these samples, in particular those in ADE, were more abundant than those detected by using the bacteria-specific primers.

Bacteria that were detected in ADE were those that have frequently been detected in ADs in previous studies [23,24], including *Clostridiaceae*, *Porphylomonadaceae*, and *Bacteroidales* (Figure 4a). Compared with bacteria that were detected in ADE, those that were increased in MFC samples were considered to play important roles in MFCs. In addition, those detected more abundantly in the AB samples than those in the PM samples were considered to be involved in power generation in MFCs. It was shown that bacterial families that increased in MFCs included *Lactobacillaceae*, *Pseudomonadaceae*, *Anaerolinaceae*, *Desulfomonadaceae*, *Pelobacteraceae*, *Peptococcaceae*, *Alcaligenaceae* and *Desulfobulbaceae* (Figure 4b). On the other hand, bacterial families that were more abundantly detected in the AB samples than those in the PM samples included *Porphyromonadaceae*, *Anaerolinaceae*, *Lactobacillaceae*, *Pelobacteraceae*, *Desulfuromonadaceae*, *Campylobacteraceae*, *Desulfobulbaceae*, *Rikenellaceae* and *Deferribacteraceae* (Figure 4b). Since these bacteria are considered important for power generation in MFC, trends in their read numbers in different samples are summarized in Table 1.

It was also noteworthy that the HRT affected the relative abundances of some bacterial families in the AB and PM fractions. For instance, *Bacteria* WM88, *Deltaproteobacteria* GW-28, *Lactobacillaceae* and *Pseudomonadaceae* were abundantly detected in MFCs operated at an HRT of 5 days, and were relatively few at the other HRTs (Figure 4b). In contrast, some bacterial families were more abundantly detected in MFCs at an HRT of 20 days than those at the other HRTs; these included *Desulfuromonadaceae*, *Pelobacteraceae*, *Syntrophomonadaceae*, *Alcaligenaceae* and *Bacillaceae*.

## 4. Discussion

The present study examined MFCs for the treatment of, and power generation from, ADE discharged from a commercial AD plant treating food wastes. It was found that MFC was able to generate power from ADE at power densities of 200 to 400 mW m^−2^ (based on the projected area of anode) and Coulombic efficiencies of 20 to 40%. Although MFC performances are affected by the reactor design, e.g., electrode area/reactor volume ratio and anode area/cathode area ratio, and operational conditions, e.g., OLR, *R*_ext_, ionic strength of electrolyte and organic substrates for power generation, the values reported in this work were considered to be relatively high compared with those for MFCs treating real ADE reported elsewhere [14]. This finding suggests that power is recoverable from ADE, if MFC is equipped downstream of AD, while further efforts are needed for improving MFCs for more efficient power recovery from ADE.

In AD plants, in particular those situated in urban areas, costs and energy are needed for treating ADE [7]. In some typical AD plants, ADE is dehydrated by pressing and/or centrifugation, and soluble fractions are treated by downstream wastewater treatment facilities, such as activated-sludge tanks and/or aerobic membrane bioreactors, before it is discharged into a sewer [7]. In order to discharge treated ADE into a sewer, environmental regulations for water qualities, such as BOD/COD, TN and TP, must be kept. The present work, therefore, assessed sCOD, sTN and sTP, in addition to tCOD, tTN and tTP, for evaluating whether MFC is suited to the treatment of ADE. Since previous studies have reported only the removal of organics (BOD and/or COD) by the MFC treatment of ADE [12,13,14,15], the data reported herein for the removal of TN and TP would be useful for researchers and engineers who will consider the application of MFCs for the treatment of ADE. It is also worth reporting that HRT affects the removal of COD, TN and TP, and these data would be the bases for designing future scale-up studies. It should, however, be noted that these MFC performances would largely be affected by reactor configurations, and we expect that more efficient MFCs, such as those that are able to remove similar amounts of COD, TN and TP at smaller HRTs, will be developed in future studies.

It was unexpected for us that, in addition to COD, TN and TP in ADE were also significantly decreased by the MFC treatment (Figure 3). In particular, as described above, the finding that sCOD, sTN and sTP can be decreased by the MFC treatment is important. In terms of sTN, previous studies have indicated that most water-soluble nitrogen species in ADE are ammonia/ammonium ion, and its excess accumulation in AD tanks results in the inhibition of methanogenic microbes and the failure in methane-gas emission [25]. We, therefore, consider that sTN in ADE used in the present study would also mainly consist of ammonia, and it is important to consider how ammonia is removed from the soluble fractions in ADE. Several previous studies have reported nitrogen removal in MFCs [26,27], and mechanisms for ammonia removal in single-chamber MFCs have been discussed elsewhere [28]. It has been suggested that nitrifying bacteria, including ammonia-oxidizing bacteria, such as those affiliated with *Nitrosomonadacea*, and nitrite-oxidizing bacteria, such as those affiliated with *Nitrospiraceae*, are present in MFCs and oxidize ammonia to nitrate with the expense of oxygen that diffuses into MFCs through air cathodes [29]. In addition, nitrate can be reduced to nitrite by heterotrophic denitrifiers, such as *Rhodocyclaceae* and *Comamonadaceae*, followed by further reduction to nitrogen gas by nitrite-reducing bacterial families, such as facultative heterotrophic *Ignavibacteriaceae* and anaerobic autotrophs, e.g., *Brocadiaceae* [29]. Although these bacteria families were not detected in abundance from the AB and PM samples, similar semi-aerobic mechanisms may have worked in the MFCs operated in this study.

In the present work, the removal of phosphorus from ADE by the MFC treatment was also observed (Figure 3). Previous studies have analyzed phosphorus species in ADE, showing that soluble phosphorus in ADE mainly consists of polyphosphates and organic phosphorus [29]. In addition, another study has detected some other reduced phosphorus species, such as phosphine and phosphite in soluble fractions in ADE [30]. Although the present work did not analyze phosphorus species in ADE and MFCE, it is likely that these water-soluble phosphorus species were removed from the soluble fractions in MFCE in the HRT-dependent manner, some of which were incorporated into sludge. In particular, since an abundantly detected bacterial group, *Deltaproteobacteria* GW-28 [31], has been known to include bacteria that perform dissimilatory phosphite oxidation to produce phosphate, it is considered that ADE contains reduced inorganic phosphorous species, e.g., phosphite, which is incorporated into sludge after it is oxidized to phosphate. In addition, albeit not large percentages, tTP in ADE was also decreased by the MFC treatment (Figure 3c). Previous studies have suggested that phosphate is chemically precipitated at the surface of the cathode [32], some of which forms struvite, a phosphate mineral containing phosphate, ammonia and magnesium [33]. Since it is conceivable that ADE contains substantial amounts of ammonia (as described above) and magnesium (that is derived from solubilized food wastes), the precipitation of phosphate as struvite in MFC would be possible.

Some bacterial families were detected more abundantly in the AB fractions than those in the PM fractions (Figure 4b, Table 1), and these are considered to be involved in power generation. For instance, *Porphyromonadaceae* is one of such families, and previous studies have detected bacteria belonging to this family in MFCs treating model biomass wastes, synthetic wastewater and pig slurry [34,35,36] as core members, and a bacterium affiliated with this family has been isolated from MFC [37]. One study has suggested a possibility that *Porphyromonadaceae* includes electrochemically active bacteria that secrete mediator compounds for electron transfer to electrodes [35]. It is, therefore, likely that bacteria in this family contribute to electricity generation in ADE-treating MFCs. *Lactobacillaceae* is a representative family for lactic-acid bacteria [38], and studies have demonstrated that some members in this family are electrochemically active [39]. To cite an instance, a study has used *Lactobacillus pentosus* for investigating reactor design and operating parameters of single-chamber MFCs for increasing power outputs [39]. We, therefore, assume that members of this family also contribute to power generation in ADE-treating MFCs.

As shown in Table 1, *Pelobacteraceae* and *Desulfuromonadaceae* were highly enriched in the AB fractions compared with those in the PM fractions. These are families in the order *Desulfuromonadales* and are closely related to the family *Geobacteraceae* that includes representative electrochemically active bacteria (EAB), such as *Geobacter sulfurreducens* [40]. Some members of *Pelobacteraceae* and *Desulfuromonadaceae* are also known to include EAB, such as *Desulfuromonas acetoxidans* [41], and they have frequently been detected from anodes in MFCs [42,43]. For instance, Miyahara et al. analyzed microbial communities established on anodes in MFCs inoculated with brackish sediments [43], showing that NaCl concentrations in media affect EAB grown on anodes. They suggested that *Geobacteraceae* bacteria grow abundantly at NaCl concentrations below 0.1 M, while *Desulfuromonadaceae* bacteria grow from 0.3 M to 0.6 M [43]. Another study showed that *Pelobacteraceae* bacteria occur abundantly in MFCs that generate power from mixed fatty acids [42]. We consider that these bacteria generate power in ADE-treating MFCs by using fermentation products, such as acetic acid. It is also important to note that *Desulfuromonadaceae* and *Pelobacteraceae* were more abundantly detected at an HRT of 20 days (Table 1). Since the higher *P*_max_ and *C*_E_ values were recorded for MFCs at an HRT of 20 days than those at HRTs of 5 and 10 days, the trends of their abundance support the idea that they are involved in power generation in MFCs treating ADE. It is likely that HRT affects species of fermentative bacteria that grow at the expense of complex organic substrates in ADE, resulting in changes in fermentation products produced in MFCs and EAB that dissimilate fermentation products. In relation to this notion, it was also found that relative abundances of some bacterial families that were detected in the PM fractions also changed depending on HRT; these included *Syntrophomonadaceae* and *Alcaligenaceae* that are known to include fermentative bacteria [44,45]. It would be interesting if pathways for degradation of high-molecular weight organic matter are changed in response to HRTs. In future studies, it would be interesting to analyze fermentation products (such as short-chain fatty acids and alcohols) that are produced in ADE-treating MFCs operated at different HRTs.

*Campylobacteraceae*, *Desulfobulbaceae*, *Rikenellaceae* and *Deferribacteraceae* were also enriched in the AB fractions, suggesting that they utilize anodes for their survival in MFCs (Table 1). Among these, bacteria affiliated with *Desulfobulbaceae* have been detected from anode biofilms in MFCs treating wastewater, and their abundance was increased under electricity-generating conditions [46]. We, therefore, suggest that *Desulfobulbaceae* bacteria detected in the present study also contribute to power generation. Concerning the other three families, there has been no report on bacteria that are able to contribute to power generation in MFCs. However, since *Deferribacteraceae* has been known to include iron- and manganese-reducing bacteria, such as *Deferribacter thermophilus* [47], we suggest a possibility that those detected in our study would be EAB. We are interested in isolating bacteria affiliated with these four families from anodes in MFCs treating ADE, and examine if they are novel EAB.

## 5. Conclusions

In order to expand the use of AD plants for recovering energy from biomass wastes, the development of schemes to reduce the operating costs of AD plants has been sought. For AD plants that are situated in urban areas, cost-effective treatment of soluble fractions in ADE would be particularly important. The present study shows that MFC can remove over 50% of sCOD, sTN and sTP from ADE at an HRT of 10 days along with power generation. Since the AD plant from which ADE was obtained in this study is operated at an HRT of approximately 20 days, the volume of MFCs should be more than half that of the AD plant for over 50% removal of sCOD, sTN and sTP. In order to reduce the volume of MFC, more efficient reactors need to be developed. To this end, further studies must be conducted to improve MFC performance, such as the development of efficient electrodes, the optimization of ratios between reactor volume and electrode areas, and the use of additives that facilitate electron transfer between microbes and electrodes. Further analyses of microbes responsible for the removal of sCOD, sTN and sTP from ADE and power generation would also provide engineers with valuable information for efficient and stable operation of MFC for the treatment of ADE. For this purpose, analyses of metabolites would also provide valuable information.

## Figures and Tables

**Table 1:** Read numbers for bacterial families that were more abundantly detected in the AB fractions than those in the PM fractions.

Family	AB				PM			
	5 Days	10 Days	20 Days	Total	5 Days	10 Days	20 Days	Total
*Porphyromonadaceae*	7936	2198	6756	16,890	2145	1868	2178	6191
*Anaerolinaceae*	1764	1698	1351	4813	637	631	717	1968
*Lactobacillaceae*	1269	313	901	2483	261	282	288	820
*Pelobacteraceae*	123	790	1263	2176	0	0	16	16
*Desulfuromonadaceae*	461	331	799	1591	5	0	12	17
*Campylobacteraceae*	23	365	48	436	24	18	41	83
*Desulfobulbaceae*	13	245	23	281	0	0	21	21
*Rikenellaceae*	9	148	46	203	0	0	8	8
*Deferribacteraceae*	13	43	48	104	11	12	0	23
Total Read	32,196	27,691	32,520	90,601	29,272	25,885	29,422	83,261

## Data Availability

The sequence data reported in this work have been deposited in the DDBJ Sequence Read Archive database under accession number DRA015529.
